# Ozone Therapy as an Adjuvant in the Treatment of Periodontitis

**DOI:** 10.3390/jcm12227078

**Published:** 2023-11-14

**Authors:** Abdulaziz Alsakr, Khalid Gufran, Abdullah Saad Alqahtani, Mohammed Alasqah, Banna Alnufaiy, Hanadi Ghurmallah Alzahrani, Ali Ayidh Alahmari, Faisal Khaled Alhumaidani, Rakan Khaled Alhumaidani, Mishari Jameel Althobiti

**Affiliations:** 1 Department of Preventive Dental Sciences, College of Dentistry, Prince Sattam bin Abdulaziz University, Alkharj 11942, Saudi Arabia; k.syed@psau.edu.sa (K.G.); ab.alkahtani@psau.edu.sa (A.S.A.); m.alasqah@psau.edu.sa (M.A.); b.alnoufaiy@psau.edu.sa (B.A.); hg.alzahrani@psau.edu.sa (H.G.A.); 2 Dental Intern, College of Dentistry, Prince Sattam bin Abdulaziz University, Alkharj 11942, Saudi Arabia; ali3ahmari@gmail.com (A.A.A.); alhumaidani.faisal@gmail.com (F.K.A.); alhomidani.rakan@gmail.com (R.K.A.); 3 Dental Department, Prince Sultan Military Medical City, Riyadh 12233, Saudi Arabia; althobiti@gmail.com

**Keywords:** ozone therapy, periodontitis, stages and grades of periodontitis

## Abstract

The current study aimed to assess the efficacy of ozone therapy in the treatment of stage II and stage III periodontitis. This prospective split-mouth study selected patients who were diagnosed with either stage II or stage III periodontitis. All patients were treated with scaling and root-planing (SRP) on the control side and SRP with ozone therapy on the test side. Probing depth (PD), clinical attachment loss (CAL), O’Leary plaque index (PI), and bleeding on probing (BOP) scores were recorded at baseline and six weeks after the SRP treatment. A total of 46 patients were selected for this study, including 31 males and 15 females. All periodontal variables (PD, CAL, PI, and BOP) showed significant changes (*p* < 0.0001) from baseline to six weeks. Moreover, significant changes (PD = 0.0001, CAL = 0.0001, PI = 0.042 and BOP = 0.0001) were also observed between the control and test sides. Gender showed no significance on periodontal variables (*p* > 0.05) except PD on the test side (*p* = 0.030). In addition, periodontal stages and grades showed no significant changes (*p* > 0.05) in any periodontal variables on both sides. Ozone therapy significantly improves the periodontal condition compared to SRP treatment alone. However, the stages and grades of periodontitis do not influence the outcome of ozone therapy.

## 1. Introduction

Periodontitis is one of the most prevalent inflammatory diseases, affecting between 20 to 50% of people globally. Severe periodontitis affects 11.2% of people around the world [1,2,3]. Gingival bleeding and tooth mobility are the most common characteristics of periodontitis; if untreated this phenomenon could lead to loss of the tooth. There are multiple factors that cause the pathogenesis of periodontal disease; bacteria play an important role [4]. Anaerobic Gram-negative bacteria are the most hostile in the progression of periodontitis [5] and are commonly found in the subgingival plaque. Due to the rapid progression of microorganisms, professional assistance is required to remove bacterial plaque [6].

Many surgical or nonsurgical therapeutic modalities are performed to eliminate biofilm or bacterial plaque [7]. Generally, scaling and root-planing (SRP) is the most conventional treatment procedure for removing sub-gingival and supra-gingival plaque and calculus [8,9]. Nevertheless, SRP cannot completely remove the pathogenic bacteria, and residual pockets, specifically in the furcation area, interproximal area, root concavities, and areas with deeper pockets, as the instrument cannot access these areas properly [10,11,12]. Previous research has indicated that the mechanical removal of bacterial plaque in patients having a pocket depth of 5 mm and more is challenging; therefore, an additional therapeutic technique is required to increase the effects of the treatment [13,14,15]. Systemic or local antibiotics and topical antiseptics have been used as adjunct therapies along with SRP for a better outcome [16,17].

A Swiss dentist first used ozonized water as a disinfectant in 1932. Since then, ozone has been studied by the scientists in different fields [18]. Fisch (1936) [18], at first, used both gaseous and water-based ozone in his dental practice. Later on, local ozone treatment showed astonishing outcomes in different fields of medicine and general surgery [19]. In recent years, ozone has been used as a supplementary treatment along with SRP in the treatment of periodontitis, as it reduces the infections instigated by Gram-negative bacteria [17,19,20,21,22,23]. Moreover, ozone also acts as an analgesic, immune stimulant, antimicrobial agent, detoxifying, and anti-hypoxic agent [24,25]. Ozone is effective against the most common Gram-positive and Gram-negative bacteria, such as *Pseudomonas aeruginosa* and *Escherichia coli*, which are known for being resistant to antibiotics [21]. In medical sciences, ozone therapy has been studied extensively due to its physiochemical properties; moreover, it is versatile in different biomedical applications such as genitourinary, degenerative, orthopedic, and neurological disorders [20,26]. In dentistry, the application of ozone therapy is used extensively in the field of endodontics, orthodontics, and conservative dentistry, even for treating tooth sensitivity [27,28,29,30]. Due to the effectiveness of ozone therapy in reducing the microbial burden and increasing the capability of the immune system, it is eligible for use in the periodontal area [31,32].

In dentistry, ozone can be used in different forms, such as gaseous, water-based, and oil-based ozone, which is considered one of the ideal therapeutic agents [24]. In addition, ozone is easily available and cost-effective to use in the field of dentistry [21]. Previously, gaseous ozone therapy was performed alongside SRP in periodontal treatments, showing improved periodontal outcomes compared to SRP alone [19]. However, gaseous ozone is not recommended for use in the oral cavity due to safety concerns [18]. Hence, ozonized water was found to improve the metabolic process that enhances wound-healing, controls bleeding, and acts as a powerful disinfectant [33]. Therefore, the current study used water-based ozone to assess the efficacy of ozone therapy in the treatment of stage II and stage III periodontitis compared to SRP.

## 2. Materials and Methods

The current prospective study was conducted at the College of Dentistry, Prince Sattam bin Abdulaziz University. The standing committee of bioethics research (SCBR) of Prince Sattam bin Abdulaziz University approved this study protocol. Moreover, the study was conducted according to the guidelines of the Declaration of Helsinki (2013).

This study was conducted from November 2022 to April 2023. All patients who were diagnosed with stage II and stage III of periodontitis according to the world classifications of 2017 [2] during this time duration in the interns’ clinic of the College of Dentistry, Prince Sattam bin Abdulaziz University were selected for the current study. The inclusion criteria of this study were: patients of both genders with an age range of 30–70 years; and a minimum presence of 12 teeth evenly distributed in the four quadrants, diagnosed with either stage II or stage III of periodontitis. Pregnant or lactating women, patients with systemic disease, patients who had undergone any non-surgical or surgical periodontal treatment six months prior to the beginning of the study, taken an antibiotic or chemotherapeutic mouth-rinse or oral irrigation six months prior to the study period were excluded from this study. The complete protocol of the study was explained to all patients included in the study and written informed consent was obtained from them.

**Figure 1:**
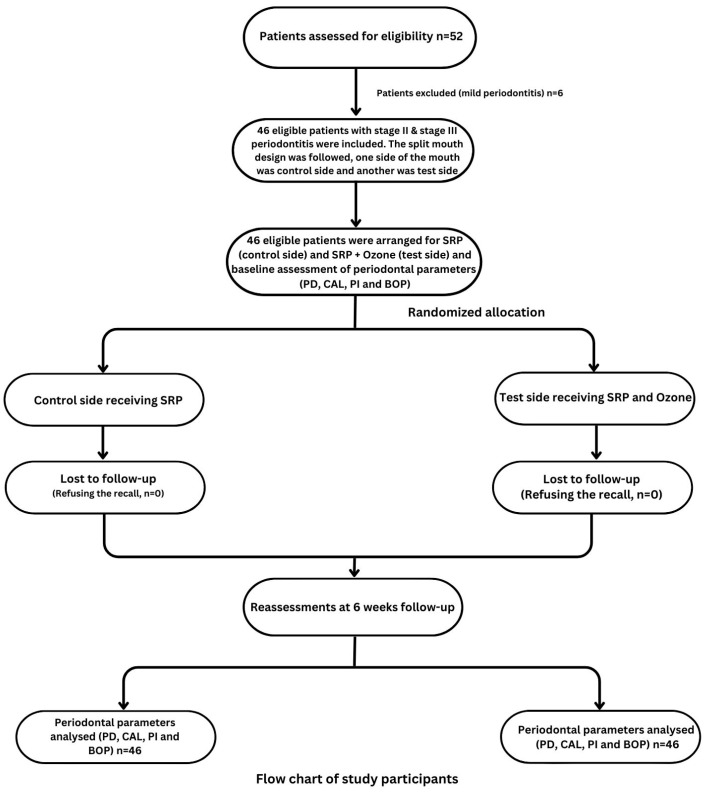
Flow chart of the study.

The split-mouth study design was followed for this current study where one side of the mouth was considered the control side, and the other was the test side. The flow chart of the study is presented in Figure 1. SRP was performed under local anesthesia on the control side and SRP with ozone therapy was performed on the test side. SRP was executed in a single visit with a minimum of 20 strokes per tooth. The Apruio3^®^ machine (Model: APL-OTM-3125, Shenzhen 518055, China) was used to obtain ozone. The ozone therapy was used immediately after the SRP using an ozone device equipped with a periodontal syringe as per the manufacturer’s instructions. The freshly generated ozone water (5–20 μg/mL) was used intrasulcularly with a sterile syringe to irrigate the periodontal pocket for 5–10 min. For irrigation, a 2 mL syringe with a 24-gauze needle was utilized. The needle was bent in the center at an angle of 110° before being inserted intrasulcularly and irrigated for 20 s.

Periodontal measurements such as the O’Leary plaque index (PI) [34], probing depth (PD) [35], clinical attachment loss (CAL) [35], and bleeding on probing (BOP) [35] were recorded at the baseline and six weeks after the SRP and ozone therapy. A total of three dental interns performed all the data collection under the supervision of the specialist periodontists and one periodontist performed SRP and the ozone application. The calibration was performed among the interns before starting the study. During the first visit, the periodontist randomly chose either the left or right side as the test side and documented this in the data collection forms, which were kept in closed envelopes. In the follow-up visits after six weeks, the examiner used a new data collection form for the same patient on which identified the right and left sides of the mouth and did not identify which side the ozone had been used. All the collected data, including the close envelopes, were sent to the statistician for data analyses. Oral hygiene instruction was provided to all patients at every visit.

Full-mouth PD was measured with the UNC 15 probe by inserting the probe parallel to the long axis of the tooth in order to grasp the deepest point of the pocket at 6 sites per tooth (mesio-buccal, mid-buccal, disto-buccal, mesio-palatal, mid-palatal, and disto-palatal), excluding the third molars. The distance between the gingival margin and the base of the pocket was recorded. The greatest probing depth was recorded for each patient.

CAL was measured from the cementoenamel junction to the apical end using the UNC 15 probe. The highest number among the CAL measurements was recorded for each patient. Moreover, a periodontal probe was inserted to the sulcus gently and swept through the proximal surface to measure the BOP. If bleeding observed after 30 min of the probe’s removal in any teeth, then the BOP was recorded.

In addition, for measuring PI, each patient was asked to chew a discoloring agent tablet thoroughly and swish the liquid around in their mouth for approximately one minute. The saliva and tablet mixture were required to coat all the tooth surfaces. Plaque was recorded in the electronic health record in the periodontal chart by recording ‘1’ if a discoloration was observed and ‘0’ where no discoloration was observed, as this meant that no plaque was present at the specific site. All surfaces were counted and calculated using the PI formula [34]:

(Plaque-free surfaces/total number of surfaces) × 100 = plaque-free score

All periodontal parameters were recorded before treatment and six weeks after the treatment.

### Statistical Analyses and Sample Size Calculation

All statistical analyses were performed using SPSS software, version 27 (IBM, Armonk, NY, USA). The normality of the data was checked using the Shapiro–Wilk test. Descriptive data were analyzed with frequency distribution. Wilcoxon signed-rank test was used to assess the difference in both sides at baseline and after six weeks. Moreover, similar statistics were also used to assess the changes of periodontal variables from baseline to 6th weeks. Mann–Whitney U test was conducted to acquire the differences in periodontal variables based on gender and stages of periodontitis. Moreover, the Kruskal–Wallis H test was performed to assess the differences in periodontal variables based on the grades of periodontitis. A linear regression model was conducted to identify the association of age, gender, the stage and grades of periodontitis with periodontal variables.

The sample size was calculated in G* power software, version 3.1. The power analysis indicated that a minimum of 34 patients were required for this study considering clinical differences of 1 mm between two treatment modalities with a 95% confidence interval and intra-group standard deviation of 2 mm with 95% power [15]. However, in order to consider the dropout rate, an additional 35% were recruited as samples for this study. Therefore, including the dropout rate, a total of 46 patients were initially selected for this study.

## 3. Results

A total of 46 patients were conveniently selected for this study including 31 males and 15 females. Shapiro–Wilk test showed that the data were not normally distributed. Therefore, non-parametric statistics were used. The descriptive data of all patients are presented in Table 1.

The Wilcoxon signed-rank test was performed to compare the periodontal variables at baseline and changes in periodontal variables from baseline to the 6th week between the control and study sides. The statistics showed that there were no significant differences in periodontal variables at baseline except CAL (*p* = 0.0001). However, all the variables showed significant differences at the 6th week (Table 2).

All periodontal variables showed significant changes (*p* < 0.0001) from baseline to the 6th week (Table 3). Moreover, significant changes were also observed between the control and study sides (Table 4). The study sides showed greater improvements in the periodontal variables compared to the control side.

The Mann–Whitney U test was conducted to compare periodontal changes between gender and stages of periodontitis. Gender showed no significance on periodontal variables except PD on the test side (*p* = 0.030) (Table 5) and periodontal stages also showed no significant changes (*p* > 0.05) in periodontal variables (Table 6). In addition, the Kruskal–Wallis H test was performed to assess the differences in periodontal variables based on grades of periodontitis, which did not show any significant changes (*p* > 0.05) in periodontal variables (Table 7).

Linear regression was conducted to identify associations between age, gender, the stages and grades of periodontitis, with periodontal variables. The results showed that age, gender, the stages and grades of periodontitis were not associated (*p* > 0.05) with the control side of any periodontal variables (Table 8). Moreover, it was observed that age (*p* = 0.009) and gender (*p* = 0.002) were significantly associated with PD on the test side. However, other variables showed no significant association on the test side (Table 9).

## 4. Discussion

The current study aimed to assess the efficacy of ozone therapy along with SRP for patients with stage II and stage III periodontitis. The study showed that ozone therapy significantly improved periodontal condition compared to the control side. However, the stages and grades of periodontitis did not show any significant differences when compared between the ozone and control group. A total of 46 patients were selected, including the dropout. However, none of the patients dropped out of this study.

When exploring the benefits of ozone, different fields in dentistry have mostly assessed the effects of ozone therapy on antimicrobial properties and wound healing [36,37,38,39,40]. Ozone therapy has also been studied in non-surgical periodontal treatments [21,23,40,41], and, like the present study, most of the studies exhibited a positive outcome [42,43,44] except a few [40,41]. However, none of the previous studies assessed the effect of ozone therapy with regard to the stages and grades of periodontitis. The current study did not find any significant difference when comparing the stages and grades of periodontitis in ozone therapy.

It is known that the colonization of pathogenic microorganisms in the subgingival and supra gingival area instigates and progresses periodontal disease. Therefore, periodontal health can be improved by removing microorganisms from the tooth’s surface. Mechanical SRP can reduce the bacterial load and biofilm around the tooth; however, only SRP alone is often not sufficient to control the progression of disease due to anatomical factors of the tooth or the invasiveness of the pathogens. In such cases, adjunctive ozone therapy could help to control the progression of the disease.

Oil-based, water-based, and air-based ozone can be used in treatments [24]. The current study used water-based ozone or ozonized water, although most studies in the literature used the gaseous form of ozone in periodontal treatments [20,21]. Even though the form of ozone usage was different, the results of our study are similar to those found in the aforementioned studies. Water-based ozone has been found to be more efficient against fungi, viruses, and bacteria as well as more cost-effective compared to alternative chemical cleansers [42]. However, research has shown that gaseous ozone is more effective against microbicides compared to aqueous ozone and it can be used as a disinfectant in dental treatment [45]. In contrast, gaseous ozone can be toxic to the respiratory tract if inhaled accidentally [44,45,46]; therefore, water-based ozone might be safer to use for the control of multiple pathogens and infections [47]. As the current study was conducted by intern dentists, the safest option of water-based ozone therapy was used.

On the other hand, in the field of endodontic and restorative dentistry, gaseous ozone is the most frequently used form of ozone. In order to avoid the inhalation of gaseous ozone, a sealing suction system is used for the topical application of ozone. The ozone is frequently used as a disinfectant in dental caries before placing restorations. Moreover, this therapy is also being used in hypo-mineralized teeth [46]. In addition, oil-based ozone is mostly used for curing fungal infections [46,48]. However, as per the literature search, no evidence was found for oil-based ozone in the field of dentistry.

The current study assessed the periodontal perimeters after six weeks of the treatment initiation. It has been mentioned in previous studies that periodontal condition after SRP should be re-evaluated at a minimum of three to four weeks after treatment. The gaining of CAL and periodontal inflammation requires three to four weeks to resolve, which also influence the probing depth. Moreover, changes in periodontal conditions occurring at one month have shown similar outcome after two and three months [49,50]. Therefore, the current study decided to follow up all patients at the sixth week, between one and two months, as no changes would occur within this period.

The current study reported that all the periodontal perimeters were significantly different after the 6th week of the treatment compared to the baseline. The median value of BOP decreased from 57.50% to 21.50% on the control side and from 57% to 10% on the study side. The median of PD was decreased from 5 mm to 4 mm and 3 mm on the control and study side, respectively. Non-surgical SRP therapy was used on both sides to effectively remove the plaque along with calculus and ensure healing, which was in agreement with the former studies [40,41]. In addition, a reduction in PI was also observed on both sides after the 6th week of treatment, which indicated the achievement of improved oral hygiene. This outcome was not unexpected as oral hygiene instructions were provided to each patient.

The baseline data of periodontal parameters were compared for both sides, and showed no significant differences except the CAL. However, the periodontal variables showed significant differences when both sides were compared at the 6th week, which was expected as the test side showed significant improvements compared to the control side. Previous studies showed that non-surgical periodontal treatment increases CAL and reduces PD in moderate to deep periodontal pockets, which is beneficial for gingival conditions [9,10,31]. However, attachment loss was also observed in shallow pockets. Therefore, it is necessary to avoid over-instrumentation. In this study, attachment loss was not observed in any patients.

Most of studies in the literature used mechanical scalers for removing the plaque and calculus [20,21,22], similar to the current study. In order to remove calculus and biofilm around the tooth, ultrasonic and hand scalers are equally effective. Additionally, a systematic review evaluated various subgingival instrumentation techniques and showed that reductions in periodontal inflammation are not dependent on the instrument used in non-surgical periodontal treatment [31]. A hand scaler was also used in a previous study with a similar study design [23]; however, the outcomes were no different from the studies using mechanical scalers.

Gender showed no significant difference in periodontal variables with the exception of PD on the study side. This might be due to the maintenance of oral hygiene. Previous studies in the literature have indicated that female patients are more careful about oral hygiene compared to male patients [51,52]. Moreover, the classification of periodontitis exhibited no significant difference among the periodontal variables on both ozone and control sides. However, outcomes of the study could not be compared directly to other previous studies, as no other study was found that assessed the stage and grades of periodontitis with ozone therapy. However, one previous study included patients with stage II and stage III periodontitis, similar to the current study, but which did not compare the outcome with the classification of periodontitis with ozone therapy [21]. Stage I periodontitis would respond to any adjunctive therapy and stage IV periodontitis requires full mouth rehabilitation. The current study was conducted in the intern’s clinic and due to complications in the management of stage IV periodontitis; stage II and stage III periodontitis were considered to be good categories for the selection of patients in this prospective study.

The positive outcome of the study suggests that ozone therapy could be a beneficial treatment not only in periodontics but also in other branches of dentistry in the future. Due to the minimal invasiveness of ozone therapy, it is of benefit to practical applications in dental treatment. However, more research should be done in future to optimize ozone therapy in different clinical practices. Even though the positive outcome of ozone therapy was demonstrated, the current study possessed some limitations. The current study assessed periodontal variables only two times, at baseline and after six weeks. The research period for intern dentists is limited, and due to the regulated time duration, the effect of ozone therapy could not be assessed over a longer period. However, a longer post-operative assessment and the application of ozone therapy multiple times could have provided more detail about the treatment protocol, and a comparison could provide, in greater detail, the more specific progression of ozone therapy. Moreover, operator limitations were not considered. As the procedure is technique-sensitive, adequate experience is required to perform it, and the results of the study could be biased based on this. A dry mouth can influence ozone treatment on the corresponding side; therefore, patients who had dry mouths, and did not report the presence of this condition, could have had an impact on the results. Therefore, further research should be conducted to overcome these limitations.

## 5. Conclusions

In conclusion, ozone therapy significantly improves the periodontal condition compared to only SRP treatment. However, the stages and grades of periodontitis do not influence the outcome of ozone therapy.

## Figures and Tables

**Table 1:** Descriptive data of the participants.

Variables		Mean	SD
Age		44.41 years	9.10
Gender [N (%)]	Male	31 (67.39)	
	Female	15 (32.61)	
Control group	BOP	62.46%	20.49
	PI	64.22%	22.72
	CAL	5.54 mm	1.06
	PD	5.02 mm	1.10
Test group	BOP	63.13%	20.65
	PI	63.78%	21.61
	CAL	6.22 mm	1.00
	PD	5.24 mm	1.49

N, total number; %, percentage; BOP, Bleeding on probing; PI, Plaque index; CAL, Clinical attachment level; PD, probing depth; SD, Standard deviation.

**Table 2:** Comparison of periodontal condition at baseline and after six weeks between test side and control side (inter group comparison).

Variables (N = 46)		Z	*p*
Baseline	BOP	−1.634	0.102
	PI	−0.049	0.961
	CAL	−4.318	0.0001 *
	PD	−1.815	0.070
After 6 weeks	BOP	−4.715	0.0001 *
	PI	−3.082	0.002 *
	CAL	−3.075	0.002 *
	PD	−5.256	0.001 *

Z, Z statistics; *p*, *p* value; BOP, Bleeding on probing; PI, Plaque index; CAL, Clinical attachment level; PD, probing depth; *, statistically significant (*p* < 0.05).

**Table 3:** Comparison of periodontal condition before treatment and after the 6th week between the control side and test side (intra-group comparison).

Variables (N = 46)		Baseline [Median (IQR)]	After 6th Week [Median (IQR)]	Z	*p*
Control side	BOP	57.50 (32.50)	21.50 (20.00)	−5.907	0.0001 *
	PI	60.00 (39.25)	20.00 (19.25)	−5.906	0.0001 *
	CAL	5.00 (1.00)	4.00 (1.00)	−5.562	0.0001 *
	PD	5.00 (1.00)	4.00 (2.00)	−5.599	0.0001 *
Test side	BOP	57.00 (30.25)	10.00 (17.00)	−5.907	0.0001 *
	PI	62.50 (31.25)	15.00 (21.25)	−5.907	0.0001 *
	CAL	6.00 (2.00)	4.00 (2.00)	−5.833	0.0001 *
	PD	5.00 (1.00)	3.00 (1.25)	−5.749	0.0001 *

N, total number; IQR, interquartile range; Z, Z statistics; *p*, *p* value; BOP, Bleeding on probing; PI, Plaque index; CAL, Clinical attachment level; PD, probing depth; *, statistically significant (*p* < 0.05).

**Table 4:** Comparison of periodontal changes over six weeks between control and test sides.

Variables (N = 46)	Control Side [Median (IQR)]	Test Side [Median (IQR)]	Z	*p*
BOP	43.50 (26.25)	50.00 (30.75)	−4.615	0.0001 *
PI	44.50 (32.25)	48 (30.75)	−2.038	0.042 *
CAL	1.00 (0.00)	2.00 (1.00)	−5.287	0.0001 *
PD	1.00 (0.00)	2.00 (1.00)	−5.488	0.0001 *

N, total number; IQR, interquartile range; Z, Z statistics; *p*, *p* value; BOP, Bleeding on probing; PI, Plaque index; CAL, Clinical attachment level; PD, probing depth; *, statistically significant (*p* < 0.05).

**Table 5:** Comparison of periodontal condition between gender and groups.

Variables (N = 46)		Z	*p*
Control side	BOP	−0.434	0.664
	PI	−0.106	0.916
	CAL	−1.183	0.237
	PD	−1.557	0.119
Test side	BOP	−0.446	0.656
	PI	−0.305	0.760
	CAL	−1.933	0.053
	PD	−2.164	0.030 *

N, total number; Z, Z statistics; *p*, *p* value; BOP, Bleeding on probing; PI, Plaque index; CAL, Clinical attachment level; PD, probing depth. *, statistically significant (*p* < 0.05).

**Table 6:** Comparison of periodontal condition between stages of periodontitis and groups.

Variables (N = 46)		Z	*p*
Control side	BOP	−0.033	0.973
	PI	−0.536	0.592
	CAL	−0.589	0.370
	PD	−0.897	0.370
Test side	BOP	−1.239	0.215
	PI	−0.045	0.964
	CAL	−1.010	0.312
	PD	−1.611	0.107

N, total number; Z, Z statistics; *p*, *p* value; BOP, Bleeding on probing; PI, Plaque index; CAL, Clinical attachment level; PD, probing depth.

**Table 7:** Comparison of periodontal condition between grades of periodontitis and groups.

Variables (N = 46)		Mean	SD	df	*p*
Control side	BOP	42.95	18.13	2	0.072
	PI	43.96	18.13		0.238
	CAL	0.96	0.63		0.676
	PD	0.93	0.61		0.567
Test side	BOP	50.48	18.79		0.098
	PI	46.95	19.81		0.601
	CAL	2.09	0.89		0.234
	PD	2.09	0.98		0.113

N, total number; Z, Z statistics; *p*, *p* value; BOP, Bleeding on probing; PI, Plaque index; CAL, Clinical attachment level; PD, probing depth.

**Table 8:** Associations between age, gender, stages, and grades of periodontitis with periodontal variables on control side.

Variables (N = 46)		Standardized Coefficient (B)	CI		*p*
			Lower	Upper	
BOP	Gender	0.034	−12.01	14.65	0.843
	Age	0.161	−0.39	1.03	0.367
	Stages	−0.071	−16.42	11.25	0.708
	Grades	0.254	−3.92	15.09	0.242
PI	Gender	0.001	−14.04	14.02	0.999
	Age	−0.092	−0.94	0.56	0.608
	Stages	0.07	−12.09	17.02	0.734
	Grades	0.026	−9.41	10.58	0.906
CAL	Gender	−0.230	−0.77	0.16	0.189
	Age	−0.101	−0.03	0.02	0.571
	Stages	0.136	−0.31	0.65	0.471
	Grades	−0.102	−0.41	0.25	0.637
PD	Gender	−0.300	−0.82	0.05	0.079
	Age	−0.196	−0.04	0.01	0.258
	Stages	0.159	−0.25	0.65	0.386
	Grades	−0.09	−0.37	0.24	0.669

N, total number; BOP, Bleeding on probing; PI, Plaque index; CAL, Clinical attachment level; PD, probing depth; CI, confidence interval; *p*, *p* value.

**Table 9:** Associations between age, gender, stages and grades of periodontitis with periodontal variables on test side.

Variables (N = 46)		Standardized Coefficient (B)	CI		*p*
			Lower	Upper	
BOP	Gender	−0.050	−15.59	11.66	0.772
	Age	0.124	−0.47	0.98	0.481
	Stages	0.151	−8.44	19.85	0.420
	Grades	0.159	−6.09	13.34	0.456
PI	Gender	−0.004	−14.92	14.56	0.980
	Age	−0.056	−0.91	0.66	0.758
	Stages	−0.057	−17.55	13.05	0.768
	Grades	0.125	−7.49	13.52	0.566
CAL	Gender	−0.314	−1.20	0.04	0.067
	Age	−0.143	−0.05	0.02	0.407
	Stages	0.089	−0.49	0.81	0.627
	Grades	−0.014	−0.46	0.43	0.948
PD	Gender	−0.474	−1.59	−0.37	0.002 *
	Age	−0.413	−0.08	−0.01	0.009 *
	Stages	0.240	−0.16	1.11	0.139
	Grades	−0.156	−0.62	0.25	0.395

N, total number; BOP, Bleeding on probing; PI, Plaque index; CAL, Clinical attachment level; PD, probing depth; CI, confidence interval; *p*, *p* value; *, statistically significant (*p* < 0.05).

## Data Availability

The datasets used and/or analyzed during the current study are available from the corresponding author upon reasonable request.
